# Predicting the Persuasiveness of Influence Strategies From Student Online Learning Behaviour Using Machine Learning Methods

**DOI:** 10.1177/07356331231178873

**Published:** 2023-06-13

**Authors:** Fidelia A. Orji, Julita Vassileva

**Affiliations:** 1 Department of Computer Science, 7235 University of Saskatchewan, Saskatoon, SK, Canada

**Keywords:** persuasive technology, persuasive strategies, machine learning, learning behaviour, persuasiveness, K-means clustering, online educational systems

## Abstract

There is a dearth of knowledge on how persuasiveness of influence strategies affects students’ behaviours when using online educational systems. Persuasiveness is a term used in describing a system’s capability to motivate desired behaviour. Most existing approaches for assessing the persuasiveness of a system are based on subjective measures (questionnaires) which are static and do not allow for automatic measurement of systems persuasiveness at run-time. Being able to automatically predict a system’s persuasiveness at run-time is essential for dynamic and continuous adaptation of the system to reflect each individual user’s state. In this study, we investigate the links between persuasiveness of influence strategies and students’ behaviour in an online educational system for a course. We implemented and tested Machine Learning (ML) classification models to determine whether persuasiveness had a significant impact on students’ usage of a learning system. Our findings revealed that students learning data can be applied to predict the persuasiveness of different influence strategies. The implications are that by using machine learning classifiers powered with learning sessions data, online educational systems would be able to automatically adapt their persuasive strategies to improve students’ engagement and learning.

## Introduction

Recent years have witnessed an increase in technological advancements focused on ways of motivating students during their learning process in both classroom and online educational systems. Some of the ways include the use of videos (Zhang et al., 2006), online educational platforms (Li & Tsai, 2017), and apps (Vazquez-Cano, 2014) to boost motivation and keep students interested in learning. These technological approaches are great ways applied to introduce some changes in teaching to continually sustain students’ motivation and make lectures not grow boring. Similarly, persuasive technologies (PTs) in education aim at the use of systems design to influence and promote students’ learning experience. Persuasive systems employ various persuasive strategies (influence strategies) to inspire and sustain users to achieve specific goals.

There is growing evidence that persuasive strategies can motivate and support students to adopt behaviours beneficial to their learning. Studies have revealed that persuasive strategies can be effective in improving students’ engagement and learning with online educational systems (Mustafa et al., 2018; Orji et al., 2019a). Persuasive strategies have been employed to design online educational systems targeted at promoting different learning processes and behaviours including improving students’ intention to perform learning behaviours (Widyasari et al., 2019) and improving self-regulated learning effort (Goh et al., 2012). The systems demonstrated the effectiveness of persuasive strategies incorporated into online educational systems at improving students’ learning behaviours. To evaluate the effectiveness of persuasive systems, the term *persuasiveness* is usually used by researchers in describing a system’s persuasive ability to motivate people for specific behaviours (Orji et al., 2019b). The persuasiveness of a persuasive strategy or system assesses its capability to inspire people to perform the desired behaviour. Online educational systems designed with persuasive strategies have the capability to change the way students interact with the systems to improve learning behaviours.

Though there is increased adoption and application of PTs in various domains, most current methods for evaluating a system’s persuasiveness rely on subjective measurements (questionnaires), which are static and do not enable automatic assessment of a system’s persuasiveness at run-time. So, there is a gap in research about methods for automatic assessments of the persuasiveness of online educational systems as students engage with the systems using objective measures (such as students’ learning behaviour). Being able to predict a system’s persuasiveness at run-time is crucial for a system to change automatically and continuously to adapt to each user’s current state. Automatic prediction of persuasiveness can be applied in adjusting or adapting influence strategies to support changes in students’ behaviours to enhance learning processes. The knowledge of when to improve persuasive effect is important for the advancement of PTs applications. Thus, this work investigated four persuasive strategies to provide empirical insight into the relationship between students’ learning behaviour and the persuasiveness of the strategies.

To explore automatic modelling of persuasiveness of influence strategies, the following research questions guide this study.

RQ1 Is there a relationship between student learning behaviour and their persuadability?

RQ2 Can persuasiveness of PT strategies be modelled automatically based on students learning behaviours?

RQ3 Which ML technique is most suitable for modelling the persuasiveness of the strategies?

This research contributes to filling the gap in existing PTs literature by utilizing ML methods and students’ learning data to develop effective models that can be applied to automatically predict the persuasiveness of PT strategies for students in an online educational system. Three important contributions are made in this research. First, using an unsupervised ML approach we show that interesting patterns revealing relationships between students learning behaviours and their receptiveness to PT strategies can be discovered. Second, using ML methods, we demonstrated that effective classification models that use learning data can be developed for predicting the persuasiveness of the strategies with high accuracy. Third, to obtain the most suitable model for predicting the persuasiveness of the strategies, the accuracy of six ML models is compared using standard performance metrics. Overall, this research gives designers of online educational systems and educators insights into how information extracted from students learning data could reveal their susceptibility to persuasive strategies which can be utilized for promoting student engagement and, potentially, the quality of teaching and learning. The following section provides an overview of related studies, followed by the ML experiment in Section 3. Section 4 explains the results and is followed by discussion and conclusion sections.

## Related Work

Research on persuasive technologies is vast, thus this review focuses on persuasive strategies, the use of learning data for prediction, and prediction of persuasiveness.

### Persuasive Strategies

Persuasive strategies are techniques employed in the design of persuasive systems to encourage users to perform desired attitudes or behaviours. Persuasive systems in education are interactive systems designed to influence students to adopt positive attitudes toward their learning processes to improve their progress. Various persuasive strategies in literature include 28 persuasive system design principles developed by Oinas-Kukkonen and Harjumaa (2009), seven persuasive strategies established by Fogg (2003), and Cialdini’s six influence principles (Cialdini, 2001). To understand and measure the capability of these strategies in motivating people to achieve desired behaviours, different tools for quantifying persuasiveness have been developed. For example, Busch et al. (2013) established a nine-point scale called the *persuadability inventory* for measuring the persuasiveness of four strategies (social comparison, reward, competition, and social learning). Similarly, a tool for assessing the persuasiveness of messages used in digital behaviour intervention in terms of effectiveness, quality, and capability was developed by Thomas et al. (2019). Kaptein et al. (2012) created the *susceptibility to persuasive strategies* scale used to assess the persuasive influence of Cialdini’s reciprocity, scarcity, authority, commitment, consensus, and liking strategies. These persuasiveness scales have been used in PT studies to provide insight into the influence of various persuasive strategies on human behaviour.

Specifically, the persuadability inventory scale (Busch et al., 2013) has been widely used in a variety of studies (Abdullahi et al., 2018; Nkwo et al., 2018; Orji et al., 2019a). The studies applied the scale in identifying users’ persuadability by different persuasive strategies so that personalized persuasive interventions could be provided to users based on the most influential strategy for them. Thus, the scale is adopted in our research in investigating the persuasiveness of the four strategies described in Table 1. These four strategies were chosen because many PT studies across domains demonstrated their efficacy in changing users’ behaviour and/or attitude positively to achieve specific tasks.

**Table 1.** Description of the Persuasive Strategies.

**Social Comparison:** The principle provides a medium for a user to observe and compare achieved performance on specific tasks to others’ performance to encourage the user to improve
**Reward:** Is based on the idea that offering users virtual incentives for performing desired tasks will improve their motivation to continue with the tasks
**Competition:** The strategy influences users by allowing them to compete with each other while performing the desired tasks
**Social learning:** Offers a user opportunity to observe the performance of other users to targeted tasks to help the user in making decisions

### Modelling With Students’ Learning Data and Prediction of Persuasiveness

The potential of achieving varied goals in research that will improve student learning progress and outcome using their learning data has been recognized. For example, studies revealed that students’ academic performance can be predicted using their learning activity to detect those who will succeed or fail so that appropriate intervention targeting weak students could be applied (Sundar, 2013). A study by Đurđević Babić (2015) demonstrated that students’ satisfaction with a course could be predicted from their learning log data. Students’ course satisfaction was assessed using a 5-point Likert scale and data from their learning logs (assignment upload, assignment view, course view, forum discussion, forum view, page view, questionnaire submit, questionnaire view, resource view, url view, user view, and user view all) were used as input variables for the models. The neural network model known as the multilayer perceptron achieved the best overall classification accuracy with an average of 73.33%. Moreover, a study showed that learning log data of students could be utilized to build models for detecting various learning affective states (such as engagement, frustration, confusion, and off-task) of students (Wang et al., 2019). The study results indicated that the correlation between affective state analysis and self-report was 83.6%, demonstrating the validity of the data analysis model. However, our research focuses on predicting the persuasiveness of PT strategies through ML methods and students’ learning data.

Often, the major obstacle in supervised ML applications is lack of labels for many real-world datasets. Supervised ML models will not be trained without labelled data. In addition, sufficient labelled data for training has to be provided for the models to achieve effective performance. Providing initial labels for real-world datasets sometimes requires special domain knowledge which might not be available or time-consuming. To derive appropriate labels for a specific dataset, exploration of patterns expressed in the dataset and partitioning of the data into similar groups using unsupervised ML techniques such as clustering have been used. For example, Gan et al. (2013) indicated that clustering is an effective knowledge discovery technique that could support training better classifiers. The researchers applied C-means clustering to unlabelled data (artificial and real datasets) to help them in training support vector machine classifier. The support vector machine model built achieved impressive performance. Furthermore, Ismail et al. (2019) applied possibilistic clustering to group visually similar image regions into clusters. The clustering process produced promising results that facilitate automatic image annotation. The researchers reported that the results of their evaluation outperform similar other solutions on automatic image annotation. The approach made it possible to accurately learn the relationship between the labels used for annotation and the visual content of the image regions.

The persuasiveness of a strategy or system estimates the capability of the strategy or system to motivate users to promote specific behaviour. However, the persuasiveness of each strategy depends on many factors, such as the domain in which it is used (e.g., e-commerce, health promotion, education) and on the individual user. To understand the influence of persuasive systems across different domains, validated persuasiveness questionnaires are used. These questionnaires allow for collecting quantitative data about users’ susceptibility to the strategies and their effect on behaviour change. They also allow tailoring the strategies and applying those that have been shown to be most effective in a specific domain. However, there is a need in research to use objective measures (such as students’ learning behaviour) obtained as students engage with persuasive educational systems to assess the persuasiveness of the systems in real-time to facilitate adjustment or adaptation of PT strategies. This need for automatic adjustment or adaptation of PT strategies has led to investigations of other methods that could predict persuasiveness dynamically. This type of approach is called implicit adaptation/personalization (Kaptein et al., 2015). For instance, different studies have utilized ML techniques to predict the persuasiveness of social multimedia communications. To discover influential factors for persuasive communication, a study (Nojavanasghari et al., 2016) explored the prediction of persuasiveness in social multimedia using a deep multimodal fusion. The researchers used the Persuasive Opinion Multimedia (POM) dataset. Data from visual, acoustic, and text modalities were combined and applied to develop a model which effectively predicted persuasiveness with an accuracy of 90%. The study demonstrated that using a deep neural network for performing late fusion produced a model with better performance for predicting persuasiveness than all previous methods applied to the same dataset. Similarly, research investigated the use of computational descriptors from visual, acoustic, and verb (such as eye gaze, head movement, voice qualities, speech disturbance ratio, etc.) in predicting persuasiveness of communication modalities on online social multimedia websites and how attributes such as user expertise could be modelled and used for predicting persuasiveness (Park et al., 2016). The research demonstrated that high-level attributes of speakers relating to persuasion such as credibility or expertise could be utilized in predicting the level of persuasiveness. Some researchers (Yang et al., 2019) proposed a neural network model that could be applied to quantify persuasiveness and detect persuasive strategies. The persuasive strategies operationalized by the authors include commitment, concreteness, emotion, identity, impact/value, and scarcity. The results of the study analysis with two datasets revealed that the effect of some persuasive strategies (identity and impact) is consistent across the datasets, however, the effects of scarcity and commitment differ, and thus caution should be applied in employing the two for diverse settings. These studies applied ML to demonstrate the feasibility of the approach in assessing persuasiveness levels. Most of the studies were conducted in the area of social multimedia to evaluate persuasive communications. To advance research on automatic prediction of persuasiveness in the online learning domain, our present study contributes to filling a gap in existing literature by developing effective models for predicting persuasiveness of PT strategies. So far, to the best of our knowledge the use of students’ learning data for predicting the persuasiveness of the strategies examined in this study using ML approach has not been explored. In addition, the input variables (total time spent, logins, assignments completed, assessment grade, and midterm exam grade) in the dataset used for this research and their interdependency were not considered in the previous studies.

## Data Collection and Preprocessing

The anonymized dataset used in this study is from 166 first-year undergraduate students that used an online learning support system in a biology course at the University of Saskatchewan in 2017–2018. The data was collected within a larger study approved by Behavioural Ethics Committee. The participants consist of 123 females and 43 males. Other students that participated in the study but did not use the online support system were filtered out. The Persuasibility Inventory Scale (PI) developed by Busch et al. (2013) was used for assessing the persuasiveness of four strategies we investigated in this research because its validity and reliability were reported by the developer and the scale has been used often in the area of persuasive technology (Abdullahi et al., 2018; Orji et al., 2019a). The questions in the scale were adapted to suit the education domain and were used to measure the susceptibility of students to four persuasive strategies: Social Comparison, Reward, Competition, and Social Learning. No question was added or removed from Busch et al. (2013) scale, but the questions were rephrased to reflect the context. The Busch’s scale includes: (1) six questions for accessing social comparison strategy – e.g., “I like to compare myself to other people” was adapted to reflect the domain as “I like comparing my academic performance against other students in a course”; (2) five questions for measuring competition strategy – e.g., “I would like to participate in Quiz show, where I need to assert myself against other people” was adapted as “I would like to participate in competitions where I’d need to challenge other students in my class”; (3) six questions for measuring reward – e.g., “it is important to me that my actions are rewarded” was modified as “it is important to me that my efforts in courses are rewarded with good grades”; (4) five questions for measuring social learning – e.g., “I adopt my behaviour quick to the model of other people” was modified as “I adopt my studying pattern quickly to the model of other students”. The questions were measured using participant agreement on a 9-point Likert scale.

### Data Preprocessing

Dataset of students’ learning logs downloaded from their learning system was preprocessed for analysis. We used Python to filter out irrelevant features and logs of students that were not part of the study from the dataset. We selected some relevant features from the dataset and they include the total time a student spent learning with the system throughout the duration of the course (Total time), the number of completed assessments out of the 12 required for the course (Assessments completed), the number of times students logged in to the learning system (Login count), the average assessment grade for each student (Assessment grade), and the midterm grade (Midterm). Overall persuasiveness of a strategy was calculated as the mean of the respective scale items for each strategy. All the strategies were considered to be persuasive since they all received mean ratings (shown in Table 2) that were greater than the neutral value of 4.5 (indicated by the scale). Since the students rated the strategies as being persuasive, this implies that using these techniques in persuasive applications will motivate students to improve their learning behaviour. Table 2 shows the descriptive statistics of the data, including the persuasiveness of the four strategies, Social Comparison, Reward, Competition, and Social Learning measured with Busch’s scale.

**Table 2.** Descriptive Statistics of the Dataset.

Variables	Number	Mean (M)	Standard Deviation (SD)
Total time	166	12445.855	1161.324
Assessments completed	166	11.096	1.979
Login count	166	34.927	20.043
Assessment grades	166	83.729	18.085
Midterm grade	166	68.428	16.803
Social comparison	166	5.565	1.264
Reward	166	7.487	0.994
Competition	166	5.475	1.503
Social learning	166	4.984	1.347

## Persuadability Modelling Framework

**Figure 1.**
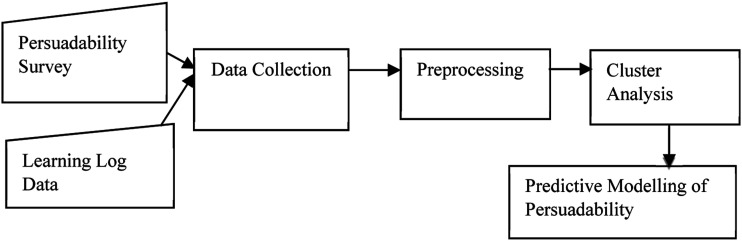
Persuadability modelling framework.

**Figure 2.**
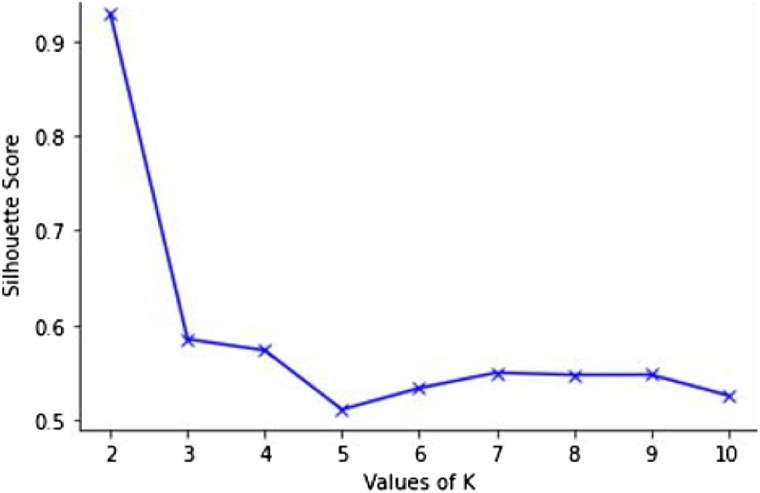
Silhouette analysis for optimal K.

We proposed an architecture for persuadability modelling framework shown in Figure 1. The framework contains two data sources – data on the susceptibility of students to some persuasive strategies assessed using Busch’s persuadability scale and the students’ interaction data from their online educational system. The data from the two sources were collected and preprocessed. The preprocessing resulted in a set of feature vectors describing specific student interaction behaviour and persuadability by each of the four strategies. All the feature vectors were continuous variables. The generated feature vectors are then categorized into groups based on their similarity using the k-means clustering technique. The learning behaviour was clustered around the measure of each strategy. We decided to employ k-means because of its simplicity, time complexity and ability to scale up nicely. To obtain the optimal k value for our dataset, we performed Silhouette Analysis (Rousseeuw, 1987). The analysis measures the compactness and separation distance between clusters and calculates the number of clusters that could yield high-quality partitions based on the cluster validity index. The silhouette score (coefficients) ranges from −1 to 1, where a high value indicates that each point in one cluster is far away from the neighbouring clusters. In order to determine the number of clusters, we used the silhouette method to analyze the data. The best silhouette scores for the strategies range from .8 to .9. An example showing one of the silhouette plots is shown in Figure 2. We choose k = 2 because it has the highest value and there is a significant drop in silhouette score between k = 2 and 3. Each group/cluster represents students that have similar persuadability and learning behaviours. The clusters will help to understand the link between student persuadability and interaction behaviour and this could assist to improve the design of online education systems. The generated clusters were analyzed to determine their characteristics and Welch’s analysis for unequal sample variances was performed to ascertain whether the clusters distinguish groups of students from each other.

According to research, a variety of techniques are available that analysts use to divide multivariate datasets into meaningful annotated partitions that are used to train ML classifiers (Chegini et al., 2019). The techniques include the use of unsupervised ML approaches (such as k-means and hierarchical clustering) which are applied to unlabelled datasets to group the data based on their similarity. The parameters of the clustering are tuned to find suitable clusters. The characteristics of the suitable clusters are utilized in training ML classifiers so that they can learn effectively the concepts expressed in a dataset. The classifiers are assessed using standard metrics to determine their capabilities at predicting future outcomes based on the information they have learned from the training set. For instance, a study applied k-means clustering to characterize the interaction behaviour of learners and the result was used to train a supervised classifier to predict successful and unsuccessful groups of learners (Amershi & Conati, 2009). Also, several studies employed clustering in discovering knowledge to improve the training of classifiers (Gan et al., 2013; Ismail et al., 2019). Based on previous studies that have applied the clustering technique and used the results to train classifiers, our framework employed the clusters identified to train supervised classifiers. The classes in the classifiers are cluster ids which are represented in binary transformation.

## Creation of Persuadability Models

Supervised classifiers have been extensively utilized in the education domain for building various leaner models for specific purposes. The classifiers require labelled data to describe the underlying structure of a dataset for training. However, labelling data needs a lot of expertise and takes time. Thus, clustering unlabelled data is valuable for training classifiers to learn the underlying structure of a dataset. A trained classifier is applied to categorize unlabelled data into already established classes.

Models for each persuasive strategy were developed separately using standard classifiers, Random Forest (RF), Logistic Regression (LR), Support Vector Machine (SVM), K-Nearest Neighbors (KNN), Decision Tree (DT), and GaussianNB (GNB). For optimizing the performance of the classifiers, hyperparameter tuning for RF, SVM, KNN, and DT was performed on the training set using the cross-validated grid search available in scikit-learn^1^ library in Python. Building models for each strategy allows the features to be optimized for that particular strategy. Multiple models were created so that the best-performing model can be identified. We consider these classifiers because they are well-known prediction models which are commonly used for building different students’ models. We used the random oversampling technique (Seiffert et al., 2010) and implemented it with the resample library in the scikit-learn package to address the imbalance problem of our dataset since an unbalanced class distribution affects the learning process of classifiers during modelling. For the modelling process, the scikit-learn library was used to implement the six classifiers for this experiment. In building our models, a percentage split was applied to randomly split our dataset into training (80%) and testing (20%) sets. The models were validated using 5-fold cross-validation at the student level. To assess the effectiveness of our feature vectors and the developed models for predicting the persuasiveness of the strategies, the models’ performance were evaluated based on the test sets using standard metrics (such as F1, accuracy, precision, and recall).

## Results

The results of our clustering analysis which explored the relationship between susceptibility of students to persuasive strategies and their learning behaviours are presented first followed by the results of our classifiers that explored the possibility of predicting the persuasiveness of the strategies from students’ learning data.

### Relationship Between Students’ Behaviour and Their Persuadability

The clustering process ensures that each student is assigned to a cluster which reduces the gap between the student characteristics and the cluster centroid. Based on the trend we observed between **cluster 1** and **cluster 2**, a descriptive label was assigned to each cluster. C**luster 1** has students that continuously use the learning system and with high persuadability scores, the cluster was labelled as *high persuadability group* (HP). The second cluster (**cluster 2**) labelled *low persuadability group* (LP) consists of students with low persuadability scores who were not consistent in using the learning system. Analysis of the two clusters revealed significant effects of clustering on all the features. The characteristics of the clusters and their analysis results are shown in Table 3. Statistically significant differences along each feature dimension exist when *p < .05* and are indicated with **. The results indicated that HP and LP clusters distinguish students in terms of learning behaviour and persuadability. The results from the clustering analysis provide insights into the relationship between students’ learning behaviours and persuadability by the PT strategies. Though there were different patterns of learning behaviours among students, it was possible to categorize students into effective groups. The k-means applied in this research characterized the students based on the statistical similarity of their data.

**Table 3.** Results of Welch’s Test for Comparison of HP and LP Clusters.

Features	HP (Mean)	LP (Mean)	F	*df*	*P<*
Social comparison	5.70	4.42	5.088	1, 86.2	.001**
Cluster members	113	53			
Total time	1347.07	863.89	22.286	1, 140.3	.001**
Assessments completed	11.65	9.85	16.523	1, 55.5	.001**
Login count	39.69	23.00	62.827	1, 152.6	.001**
Assessment grades	91.29	67.60	54.765	1, 58.0	.001**
Midterm grade	75.47	52.60	123.388	1, 92.2	.001**
Reward	7.81	4.50	29.310	1, 18.1	.001**
Cluster members	101	65			
Total time	1370.78	916.25	19.695	1, 163.6	.001**
Assessments completed	11.07	10.23	13.368	1, 71.7	.001*
Login count	40.64	24.60	55.957	1, 163.5	.001**
Assessment grades	90.86	72.65	38.240	1, 77.1	.001**
Midterm grade	77.01	54.43	144.629	1, 125.9	.001**
Competition	5.66	4.45	5.582	1, 112.7	.001**
Cluster members	111	55			
Total time	1313.34	949.53	10.514	1, 1222.2	.002**
Assessments completed	11.67	9.87	17.513	1, 57.6	.001**
Login count	39.42	24.14	48.646	1, 151.8	.001**
Assessment grades	91.55	67.95	57.187	1, 59.8	.001**
Midterm grade	76.01	52.32	146.597	1, 97.3	.001**
Social learning	4.98	4.34	.019	1, 110.1	.021**
Cluster members	110	56			
Total time	1318.80	945.30	11.255	1, 126.4	.001**
Assessments completed	11.69	9.86	18.780	1, 58.5	.001**
Login count	39.58	24.11	50.088	1, 153.5	.001**
Assessment grades	91.72	68.04	59.571	1, 60.8	.001**
Midterm grade	76.09	52.60	142.379	1, 97.8	.001**

### Model Prediction

The results of the models’ accuracy scores are based on the test set for the four strategies. The accuracy scores of the classifiers for the strategies are presented in Tables 4–7. On average, the tested models achieved considerable performance in predicting the persuasiveness of the four strategies. Interestingly, the Decision Trees (DT) models achieved the highest accuracy scores (in terms of accuracy, precision, recall, and F1) for all of the four persuasive strategies, which suggests that among the ML methods investigated, the DT models are the most suitable for predicting persuasiveness to the strategies using learning data. The DT models’ F1 scores for the strategies range from 77% to 92%. The RF model with F1 scores ranging from 75% to 88% was the second-best performing model. The models can be applied in detecting students with low and high persuadability to a persuasive strategy. These results show that the learning behaviours of students are sufficient to predict their persuadability by the strategies considered in this research.

**Table 4.** Models Prediction Performance for the Persuasiveness of **Social Comparison Strategy**.

Metrics	RF	LR	SVM	KNN	DT	GNB
Accuracy	0.88	0.60	0.61	0.68	**0.92**	0.54
Precision	0.88	0.61	0.71	0.67	**0.93**	0.40
Recall	0.87	0.60	0.58	0.67	**0.91**	0.09
F1	0.88	0.60	0.51	0.67	**0.92**	0.15

**Table 5.** Models Prediction Performance for the Persuasiveness of **Reward Strategy**.

Metrics	RF	LR	SVM	KNN	DT	GNB
Accuracy	0.85	0.63	0.47	0.58	**0.91**	0.54
Precision	0.85	0.66	0.73	0.62	**0.91**	0.75
Recall	0.85	0.65	0.53	0.61	**0.91**	0.25
F1	0.85	0.63	0.37	0.58	**0.91**	0.38

**Table 6.** Models Prediction Performance for the Persuasiveness of **Competition Strategy**.

Metrics	RF	LR	SVM	KNN	DT	GNB
Accuracy	0.82	0.71	0.52	0.73	**0.87**	0.69
Precision	0.82	0.71	0.64	0.73	**0.87**	0.66
Recall	0.83	0.71	0.54	0.72	**0.87**	0.72
F1	0.82	0.71	0.43	0.72	**0.87**	0.69

**Table 7.** Models Prediction Performance for the Persuasiveness of **Social Learning Strategy**.

Metrics	RF	LR	SVM	KNN	DT	GNB
Accuracy	0.76	0.60	0.41	0.64	**0.78**	0.69
Precision	0.75	0.62	0.21	0.63	**0.77**	0.67
Recall	0.75	0.62	0.50	0.63	**0.77**	0.50
F1	0.75	0.60	0.29	0.63	**0.77**	0.57

### Features Significant for the Strategies

**Figure 3.**
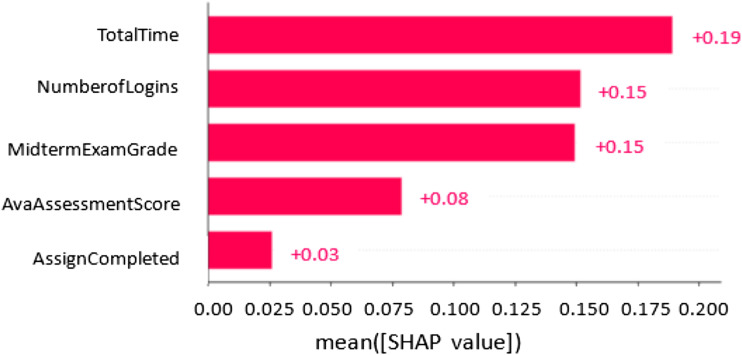
SHAP value showing the impact of the features on model output.

To understand and explain the outputs of our ML classifiers, we employed a popular Explainable AI framework called Shapley Additive exPlanation (SHAP) (Lundberg et al., 2017). SHAP applies mathematical methods to aid in understanding and interpreting output of ML models. It provides information which helps in understanding the relationship between model prediction and components of feature vectors applied to generate the prediction. The SHAP method provides values (called SHAP values) that measure feature importance to explain the impacts of each feature on a model. Figure 3 shows the contribution of each feature based on the SHAP values. The order of the features in the figure is based on their influence on the model prediction. The SHAP framework provides insights about the process that is being represented and how a model could be improved.

Based on Figure 3, all the features contributed positively to the models’ predictions. The most predictive feature among the three strategies (competition, social comparison, and social learning) is time on task. Spending more time in the educational system aids students to accomplish their learning tasks in order to achieve performance expectations. For reward strategy, the most predictive feature is the number of logins. In reward-based educational systems, students can login frequently to the system and perform small tasks to gain rewards. However, the least predictive feature of the four strategies is the number of assessments completed. Thus, spending more time on an online educational system has the highest impact on our model prediction.

## Discussion

This paper presented the results of ML experiments investigating the possibility of creating effective models for predicting the persuasiveness of PT strategies using students learning data. It also provides insight into the use of clustering analysis as a pre-processing step for classification problems. The clustering analysis was performed to determine whether natural grouping that will improve the classification process could be identified. Based on the results of Welch’s analysis for unequal sample variances shown in Table 3, the HP clusters were significantly different from LP clusters for all the strategies. In other words, higher and lower susceptibility to persuasive strategies seems to manifest itself in different behaviour of students. The clusters discovered students with similar learning behaviours and persuadability as exemplified by the behavioural difference between the clusters. So the Total Time, Assessments Completed, Login Count, Assessment Grades, Midterm Grade and Persuadability scores tend to be higher in the HP cluster and lower in LP clusters. This suggests that the clustering process was able to learn effectively the association between students’ persuadability and learning behaviour. The HP clusters contain more students. This seems to suggest that the different strategies are appropriate for most of the students. For our RQ1, the findings from the cluster analysis showed that associations exist between student learning behaviour and persuadability. The findings help in determining high and low persuadability which were used as a basis for the modelling process.

To answer research questions RQ2 and RQ3, findings from this study revealed that learning behaviour data can be effectively used for modelling the persuasiveness of the strategies. Two (random forest and decision tree) of the six different ML classifiers implemented to investigate the persuasiveness modelling achieved good prediction performance (in terms of *F1, Accuracy, Precision,* and *Recall*) on the test set for all the strategies. F1 scores of random forest for all the strategies range from 75% to 88%. More importantly, the decision tree (DT) classifier produced significant prediction models for all the strategies studied, achieving F1 scores of between 77% and 92%. The performance of the models varied across the strategies with scores of 92%, 91%, 87%, and 77% for social comparison, reward, competition, and social learning respectively. This suggests that the persuasiveness of some strategies can be more effectively inferred from learning data than that of others. Overall, models with high F1 scores above 85% were produced, showing that successful classification models for predicting students’ persuadability could be constructed based on their learning behaviour in a course, which answers the second research question, RQ2. This implies that students’ persuadability could be automatically discovered in online educational systems based on their learning behaviour characteristics. As shown in Figure 3, all the features studied contributed positively to persuasiveness assessments, demonstrating the applicability and effectiveness of learning behaviour data for persuasiveness modelling. However, the most contributing feature is the total time spent on the online educational system followed by the number of logins and midterm exam. The generic activity features used in the models are likely to generalize to other learning domains. This study has demonstrated and assessed the proposed framework and identified the impacts of the features in persuasiveness modelling.

Several of our findings are consistent with prior studies that detected some students learning attributes using their learning data. For instance, Đurđević Babić (2015) reported that neural network model with an accuracy score of 73.33% is the best model for predicting students’ satisfaction in a course using their learning data. Wang et al. (2019) revealed that students’ learning affective states such as concentration, frustration, confusion, and off-task could be predicted from their learning data with F-scores of 57%, 74%, 44%, and 75% respectively using Nave Bayes and K* algorithms. Moreover, Park et al. (2016) applied multimodal models and predicted the persuasiveness of communication modalities on social multimedia with a mean accuracy score of 70.34%. As a comparison, our persuasiveness prediction models achieved accuracy scores of 92% for the social comparison strategy, 91% for the reward strategy, 87% for the competition strategy and 78% for the social learning strategy. The accuracy is better than comparable studies in the literature. Even so, the key would be designing intervention strategies that are effective when the diagnosis is accurate and not de-motivating when the diagnosis is inaccurate. Most of the works on automatic detection of persuasiveness are based on social multimedia. In the online education context, ML models built with learner interaction data have been demonstrated to successfully predict learner engagement, affective states, and satisfaction with a course. Thus, the current findings establish a benchmark for what can be accomplished when the learning behaviour data of students are utilized to predict the persuasiveness of four commonly used persuasive strategies.

Most importantly, currently, there have been very few, if any, attempts to personalize dynamically the persuasive strategies to the current needs of the user. Most, if not all, persuasive technology systems are designed in a “one-size-fits-all” approach or at most, use several static designs tailored to the needs of different user types, based on their highest susceptibility to one of the persuasive strategies deployed by the system. These user types are established through laboratory studies using validated surveys and statistical analysis (regression analysis, structural equation modelling, etc.). The high accuracy of predictive models we developed in this study from user behaviour data allows for personalizing persuasive strategies dynamically, on the fly, to maximize their effect on behaviour change. Below we discuss the implications of our findings and how they can be applied to improve the efficacy of persuasive educational systems.

Persuasive educational systems are designed to persuade students to achieve specific objectives, e.g. increase engagement, help establish good learning habits, increase motivation for learning, etc. Uncovering the possibility of modelling students’ persuadability by different persuasive strategies using learning data will help to improve the design of persuasive systems to make them more effective. According to research, “persuasive technologies which use implicit personalization, operative measures are used to estimate the individual susceptibility of users to influence principles” (Kaptein et al., 2015). In such implicit personalization, user interactions are used for profiling and adaptation of persuasive strategies. Thus, to aid in the dynamic and continuous adaptation of persuasive educational systems to reflect the ever-changing states of learners, there is a need to develop a method for automatically assessing the persuasiveness of systems as students engage with them using objective data of student learning behaviours. This approach of predicting persuasiveness automatically and dynamically is better than the currently dominant approach used for static adaptation, which relies on questionnaires, typically done in lab conditions, with different users. The persuadability models created in this research can be integrated into online educational systems so that the system can predict persuasiveness in real-time. The information gathered can be used in a variety of ways, starting with a deeper understanding of students’ learning experiences while using the system. Is the persuasiveness of the strategies used in the system design consistent as students continue to use it or does it drop? This will help in informing instructional design and adaptation of the strategies. Automatic detection of persuasiveness also opens the opportunity for developing interventions that will assist in regulating the persuasive effect of systems. It will help in determining when interventions should be delivered. For instance, students who show signs of low persuadability at certain points might be persuaded by increasing the intensity of the persuasive effect or by using another strategy.

## Limitations and Future Works

Though the results of this research are crucial and interesting, there are limitations. Our sample size for this study was small and our experiment was limited to one undergraduate course. We acknowledge that our data is from pre-covid era and findings may not generalize with post-covid data. For our future work, we will collect post-covid data and compare the results with our findings.

The models developed can be applied to students using other learning platforms for various courses to evaluate their generalizability. However, all the features used for our models are common among other learning platforms. Furthermore, it would be interesting to test other learning features and ensemble techniques of ML in the modelling process, and also to possibly investigate the application of the models in a personalized persuasive educational system.

## Conclusion

This research investigated the possibility of developing effective classification models for predicting the persuasiveness of PT strategies with ML techniques using students’ learning data and to identify the most suitable model among six alternatives. Before performing the modelling process, the association among all predictors and target variables was discovered using an unsupervised ML technique (clustering). In the modelling process, six ML techniques yielded useful prediction results regarding the students’ persuadability by four commonly used persuasive strategies (social comparison, competition, reward and social learning). Our results revealed that the Decision Tree model outperforms the other models as it produced the highest overall classification accuracy scores for all four strategies. The results imply that the persuadability of students to persuasive strategies can be modelled from their digital traces in online educational systems (in our case - logins, total time spent, assessments completed, assessment grades, and mid-term exam grades). Predicting automatically students’ persuadability is a necessary step in adapting persuasive strategies or moderating their impact. This knowledge will be necessary for developing persuasive learning interventions for improving students’ learning experience and outcomes.

Though this research used learning variables and different ML algorithms in modelling students’ persuadability by PT strategies, the results obtained are in line with other studies which modelled various students’ learning attributes using learning data, previous results in other application domains, and confirm that ML techniques can generate useful models for predicting the persuasiveness of PT strategies.

## Note

1.
https://scikit-learn.org
